# Supplementary material for: The DUF348 domains of resuscitation promoting factor 2 play important roles in the enzymatic and biological activities in Rhodococcus erythropolis KB1
Source: PeerJ. 2024 Nov 19;12:e18561. doi: 10.7717/peerj.18561 (PMC11583912; doi:10.7717/peerj.18561)
Supplement: Supplemental Information 1 [file peerj-12-18561-s001.doc]

| **protein name** | **molecular weight** | **amino acid sequence** |
| --- | --- | --- |
| Rpf2 | 39.04kDa | MSPFAKINSTKSPLLYSVVGALLLTLIVGGALAVVRHKTITLDVDGDMISLSTMSSSVGTALEDAGYPVDPKDALAPSADSSLSDGDTVVLRRARELALTVDGQPTTIWTTALTVDEALKQVDVSDDFAVSASRSHRLPLERTELDVVSPKTVSVSDGGAPAAEIKLAAVTVGELLAAKNAPLEQADSVVPTADTPVTNGLTIQVTRDRTDTRVETQPIAPPENKVEDPELEKDKTVVENPGVAGERTATVEVKTVNGVEAGRTELSATVIKEPVAAVVKVGTKQTSAPAVSNASTWDSIAQCEATGNWAINTGNGFYGGLQFTQSTWEAFGGGQYAARADLASREQQIAIGEKVRAGQGWGAWPSCTSKLGLR |
| Rpf2 lacks 1 DUF348 domain | 35.41kDa | HMVSPFAKINSTKSPLLYSVVGALLLTLIVGGALAVVRHKTSSLSDGDTVVLRRARELALTVDGQPTTIWTTALTVDEALKQVDVSDDFAVSASRSHRLPLERTELDVVSPKTVSVSDGGAPAAEVKLAAVTVGDLLAAKNTPLEQADSVVPTAETPVTNGLTIQVTRDRTDTRVETQPIAPPENKVEDPELEKDKTVVENPGVPGERTATVEVKTVNGVEAGRTELSATVIKEPVAAVVKVGTKQASAPAVSNASTWDAIAQCEATGNWAINTGNGFYGGLQFTQSTWEAFGGGQYAARADLASREQQIAIGEKVRAGQGWGAWPSCTSKLGLRLE |
| Rpf2 lacks 2 DUF348 domain | 32.52kDa | HMVSPFAKINSTKSPLLYSVVGALLLTLIVGGALAVVRHKTSSLSDGDTVVLRRARELADDFAVSASRSHRLPLERTELDVVSPKTVSVSDGGAPAAEVKLAAVTVGDLLAAKNTPLEQADSVVPTAETPVTNGLTIQVTRDRTDTRVETQPIAPPENKVEDPELEKDKTVVENPGVPGERTATVEVKTVNGVEAGRTELSATVIKEPVAAVVKVGTKQASAPAVSNASTWDAIAQCEATGNWAINTGNGFYGGLQFTQSTWEAFGGGQYAARADLASREQQIAIGEKVRAGQGWGAWPSCTSKLGLRLE |
| Rpf2 lacks 3 DUF348 domain | 28.61 kDa | HMVSPFAKINSTKSPLLYSVVGALLLTLIVGGALAVVRHKTSSLSDGDTVVLRRARELADDFAVSASRSHRLPLERTELDVVSPKTETPVTNGLTIQVTRDRTDTRVETQPIAPPENKVEDPELEKDKTVVENPGVPGERTATVEVKTVNGVEAGRTELSATVIKEPVAAVVKVGTKQASAPAVSNASTWDAIAQCEATGNWAINTGNGFYGGLQFTQSTWEAFGGGQYAARADLASREQQIAIGEKVRAGQGWGAWPSCTSKLGLRLE |

Table S1The wild-type Rpf2 and deletion of DUF348 domain Rpf2 protein sequence and molecular weight
