# Supplementary material for: The DUF348 domains of resuscitation promoting factor 2 play important roles in the enzymatic and biological activities in Rhodococcus erythropolis KB1
Source: PeerJ. 2024 Nov 19;12:e18561. doi: 10.7717/peerj.18561 (PMC11583912; doi:10.7717/peerj.18561)
Supplement: Supplemental Information 2 [file peerj-12-18561-s002.doc]

| Protein | pTM score |
| --- | --- |
| Rpf2 | 0.57 |
| Rpf2 lacks 1 DUF348 domain | 0.59 |
| Rpf2 lacks 2 DUF348 domain | 0.53 |
| Rpf2 lacks 3 DUF348 domain | 0.45 |

Table S2 pTM score of Alphafold3
