# Supplementary material for: The DUF348 domains of resuscitation promoting factor 2 play important roles in the enzymatic and biological activities in Rhodococcus erythropolis KB1
Source: PeerJ. 2024 Nov 19;12:e18561. doi: 10.7717/peerj.18561 (PMC11583912; doi:10.7717/peerj.18561)
Supplement: Supplemental Information 3 [file peerj-12-18561-s003.doc]

Table S3 Significance test of the promoting effect of wild-type Rpf2 and variant proteins lacking different amounts of DUF348 on the growth of *R. erythropolis* KB1

| **Tukey's multiple comparisons test** | **Significant?** | **Summary** | **Adjusted P Value** |
| --- | --- | --- | --- |
| Without Rpf2 vs. Inactive Rpf2 | No | ns | 0.6657 |
| Without Rpf2 vs. Rpf2 | Yes | ** | 0.0088 |
| 1△DUF348 vs. Rpf2 | Yes | * | 0.0215 |
| 2△DUF348 vs. Rpf2 | Yes | * | 0.0262 |
| 3△DUF348 vs. Rpf2 | Yes | * | 0.0150 |
