# Supplementary material for: The DUF348 domains of resuscitation promoting factor 2 play important roles in the enzymatic and biological activities in Rhodococcus erythropolis KB1
Source: PeerJ. 2024 Nov 19;12:e18561. doi: 10.7717/peerj.18561 (PMC11583912; doi:10.7717/peerj.18561)
Supplement: Supplemental Information 4 [file peerj-12-18561-s004.doc]

Table S4 Significance test of the effect of wild-type Rpf2 and mutant proteins with different amounts of DUF348 deletion on the recovery of VBNC *R. erythropolis* KB1

| **Tukey's multiple comparisons test** | **Significant?** | **Summary** | **Adjusted P Value** |
| --- | --- | --- | --- |
| Without Rpf2 vs. Inactive Rpf2 | No | ns | >0.9999 |
| Without Rpf2 vs. Rpf2 | Yes | **** | <0.0001 |
| Rpf2 vs. 1△DUF348 | Yes | * | 0.0309 |
| Rpf2 vs. 2△DUF348 | Yes | **** | <0.0001 |
| Rpf2 vs. 3△DUF348 | Yes | **** | <0.0001 |
| 1△DUF348 vs. 2△DUF348 | Yes | * | 0.0116 |
| 1△DUF348 vs. 3△DUF348 | Yes | *** | 0.0007 |
| 2△DUF348 vs. 3△DUF348 | No | ns | 0.5138 |
